# Analyzing Occupational Performance of Children With Handwriting Difficulties: Parent and Teacher Experiences and Perspectives

**DOI:** 10.1155/oti/8882049

**Published:** 2025-02-26

**Authors:** Dini Fajariani, Natsuka Suyama, Yoko Yamanishi, Supaluck Phadsri, Dwi Ayu Komariyah, Yuko Ito

**Affiliations:** ^1^Department of Occupational Therapy, Tokyo Metropolitan University, Tokyo, Japan; ^2^Department of Occupational Therapy, University of Indonesia, Depok, Indonesia; ^3^Department of Occupational Therapy, Chiang Mai University, Chiang Mai, Thailand

**Keywords:** environment, handwriting, improvement, inhibiting factors, supportive factors

## Abstract

**Background:** Handwriting is a crucial skill for elementary school students that involves complex subskills, including visual coordination, motor planning, cognitive abilities, and self-regulation. To inform intervention plans and support occupational performance and participation, occupational therapists use occupational profiles and performance analysis to assess children's strengths, weaknesses, handwriting performance, and school and personal contexts.

**Objective:** This study is aimed at identifying and assessing the characteristics, as perceived by parents and teachers, that both facilitate and impede the improvement of children's handwriting.

**Methods:** We employed a qualitative approach with inductive content analysis and collected data via semistructured individual interviews following purposive sampling of parents and teachers (*N* = 8) in Indonesia. The initial and subsequent interviews lasted 30–60 min and 20–40 min, respectively.

**Results:** We analyzed the results based on two themes: (1) client factors that enhance handwriting abilities and (2) environmental factors that contribute to improving handwriting abilities. Psychological well-being, cognitive abilities, and motor performance significantly influenced handwriting skills. Children with poor emotional control, boredom, lack of age readiness, and memory difficulties often struggled with writing. Additionally, underdeveloped fine motor skills contributed to impaired handwriting abilities.

**Conclusions:** Our findings have significant implications for occupational therapists. It highlights the impact of motor-related and external factors on handwriting abilities in children. The study suggests that occupational therapists can tailor interventions to children's needs by understanding parents' and teachers' perspectives. Additionally, it underscores the importance of collaboration between parents, therapists, and teachers in improving handwriting skills and emphasizes the critical role of siblings and peers in enhancing children's handwriting performance.

## 1. Introduction

Handwriting is a complex skill requiring the integration of visual and motor coordination, motor planning and execution, cognitive and perceptual capabilities, and kinesthetic awareness to perform writing tasks effectively [1–3]. For elementary school students, handwriting is essential for academic activities such as noting assignments, writing observations during lessons, transcribing information from the board, and completing handwritten homework, essays, and math problems [1, 4].

The educational system in Indonesia comprises formal, nonformal, and informal education, which are designed to complement and enhance one another. Formal education includes primary, secondary, and higher education levels [5]. According to the 2024 regulation from Indonesia's Ministry of Education, Culture, Research, and Technology, children are expected to begin prewriting practice in kindergarten and achieve basic writing skills at the elementary level [6].

Teachers often seek the assistance of occupational therapists when students struggle with handwriting, which hinders their ability to complete written assignments [7]. A scoping review by Renahan et al. [8] highlights the role of occupational therapists in supporting major educational transitions for students with disabilities. These roles include assisting with student progression, participating in Individualized Education Program (IEP) meetings, collaborating interprofessionally, guiding families through transitions, and contributing to curriculum development. Similarly, Meuser et al. [9] observed that teachers and occupational therapists characterized their collaboration as a progressive journey toward mastery and trust. At advanced stages, their partnership enabled them to design, implement, and evaluate methods for improving outcomes for all children.

Research demonstrates the positive impact of occupational therapy interventions on student performance. For instance, a study of 91 school-aged children with fine motor difficulties showed improved occupational performance when teachers better understood students' needs and effectively applied occupational therapy strategies [10]. Beyond teacher collaboration, parents and therapists often work as equal partners, setting goals and implementing interventions through joint decision-making [11]. During assessment and intervention, occupational therapists focus on three primary domains: (1) handwriting analysis, identifying specific handwriting challenges (e.g., spacing or letter formation); (2) the educational environment, including curriculum and classroom setup; and (3) the student's individual circumstances, such as cultural or temporal factors [7].

The evaluation process involves understanding the client's goals and needs, assessing their abilities and achievements, and identifying factors that support or hinder their well-being and engagement. Occupational therapists analyze the occupational profile and performance, considering the dynamic relationships between occupational demands and the client's contexts, performance patterns, skills, and client factors. Evaluating these components holistically enables therapists to address barriers and enhance engagement in meaningful activities [12].

Although research on handwriting interventions exists, there is limited evidence identifying specific factors that facilitate or impede children's handwriting improvement through occupational therapy interventions. Exploring children's occupational profiles helps uncover how past experiences influence handwriting performance. Additionally, assessing occupational performance reveals interactions among contexts, performance patterns, and client factors that may hinder handwriting interventions. Understanding handwriting difficulties from the perspectives of parents and teachers is crucial [13, 14]. Therefore, this study is aimed at identifying and assessing the characteristics that facilitate and impede the improvement of children's handwriting as perceived by parents and teachers.

## 2. Methods

### 2.1. Study Design and Ethical Approval

We employed a qualitative research methodology using inductive content analysis to identify thematic patterns from an occupational perspective. The primary investigator conducted individual, semistructured interviews to gather insights into the factors that facilitate or hinder the enhancement of children's handwriting skills from parents and teachers. This study received ethical approval from the university ethics committee where the research was conducted (Approval No. 23012). All participants provided informed consent before participating.

### 2.2. Participants

The study was conducted at a private elementary school in Indonesia from October to November 2023. Five parents and three main teachers of first-grade primary school children with handwriting difficulties were identified through the school's routine screening assessment. To recruit participants, the researcher asked teachers to select first-grade students who had handwriting challenges based on the school's regular screening test. This test is administered annually before the start of the first semester. Individuals exhibiting neurological symptoms, physical disabilities, or intellectual disabilities that could affect their handwriting abilities were excluded, as were those receiving special education assistance. The parents and teachers of selected students were then contacted by the primary researcher, who explained the study and obtained their consent to participate. All invited participants agreed to take part in the study.

### 2.3. Procedures

The interviews were conducted in two separate sessions with a 1-week interval. The initial session aimed at collecting observations and viewpoints on children with handwriting challenges from parents and teachers and identifying elements that facilitate or hinder handwriting improvement. The second session aimed at validating the information discussed during the first session. Each session lasted 30–60 min for the initial interviews and 20–40 min for the follow-ups. The interviews were conducted via internet video conferencing between October and November 2023. Participants completed a brief demographic questionnaire before the interviews, which collected information such as age, occupation (for parents), and years of experience (for teachers). Semistructured interviews included five to seven primary questions designed to elicit participants' experiences and perceptions of handwriting challenges. The interview questions for parents and teachers are presented in Tables 1 and 2, respectively. All interviews were audio and video recorded, and aliases were used during transcription to ensure confidentiality.

### 2.4. Data Analysis

Data were analyzed using inductive content analysis, a methodology commonly employed when prior research on a specific issue is limited or lacks coherence. The researchers examined the data for similarities and differences, organizing them into categories and themes through varying levels of abstraction and interpretation. This process involved transforming specific data into broader, more generalized concepts [15]. For instance, the quotation, “Yes, so it's really, how should I say it, purely a problem because he just doesn't feel like writing,” was coded as “low motivation to study.” This code was then grouped into the subcategory “regulation of emotions,” which was further categorized under “child's psychological well-being.” Ultimately, it contributed to the overarching theme “client factors that enhance handwriting abilities.”

Initially, all video and audio recordings were meticulously transcribed manually. Transcripts were then reviewed against the recordings to ensure accuracy. Researchers examined each interview to gain a comprehensive understanding of the dataset. Initial concepts were outlined in written records and emphasized to understand the data fully. Sentences and paragraphs providing evidence for research topics were identified and systematically categorized. Codes were then compared and organized into emerging categories based on variations and similarities. The raw data were reassessed, reread, and structured into overarching themes. We aimed to provide a detailed explanation of the organization of thematic findings, supported by corresponding categories and relevant quotes [16]. Three researchers (D.F., S.P., and D.A.K.) analyzed the data and recorded the findings. Two researchers held master's degrees in occupational therapy (D.F. and D.A.K.), and one held a doctoral degree in the same field (S.P.).

## 3. Results

Five parents (all mothers) and three teachers participated in the study. The teachers had an average of 13 years of work experience and demonstrated a thorough understanding of their students. The findings were organized into two primary themes: (a) client factors that enhance handwriting abilities and (b) environmental factors that contribute to improving handwriting abilities. Detailed findings are presented in Table 3.

### 3.1. Theme 1: Client Factors Enhancing Handwriting Abilities

During the interviews, the participants were questioned about their first-hand knowledge and perspectives regarding the environment and factors contributing to the enhancement of handwriting skills, and data analysis revealed two distinct categories: (a) psychological well-being and (b) cognitive and motor performance. Each of these categories was then examined thoroughly.

#### 3.1.1. Psychological Well-Being

The participants emphasized that children's handwriting abilities are significantly influenced by their psychological well-being. This broad concept encompasses cognitive, emotional, and psychological aspects of a child's life, as well as their social environment. For example, a child's mood, motivation, and emotional regulation directly impact handwriting performance.

However, on occasions when he is ordered, the outcome tends to be sloppy. When he is not in the appropriate mood, he exhibits such behavior, but when he is in the suitable mood, his writing is meticulous. (P3)

Children prone to boredom often employ various excuses to avoid completing their schoolwork.

Typically, when he is feeling bored, he chooses to stand up. Then say I would like to first have a drink downstairs, followed by a snack. After that, I would like to use the restroom. (P5)

The participants also noted that age-related maturity and confidence are essential for handwriting proficiency. Immature cognitive processes can result in a lack of confidence and poor handwriting performance.

Based on my findings, one fundamental aspect is maturity, including age-related maturity. Additionally, there are individuals who possess proficient reading and writing skills but lack the self-assurance to showcase their abilities among their peers. According to my observations, one of the basic things is maturity—the maturity of the age too. There are also those whose ability to read and write is already good, but their confidence among their friends has not yet emerged. (P8)

#### 3.1.2. Cognitive and Motor Performance

Interview data indicated that memory and concentration difficulties frequently hinder children's handwriting abilities. Children often struggle to recall letter forms, leading to errors and reversals during writing.

However, after he acquires knowledge of the letters, he frequently experiences slip in memory. Frequently neglect to intentionally disregard and subsequently encounter a situation that is in an inverted or reversed state. (P2)

Additionally, children with limited sustained attention may become easily distracted during learning activities.

If the duration is thirty minutes, it is not considered complete as there are numerous excuses included. If, perhaps for certain individuals, one and a half hours seems excessively lengthy, eh, I will eat this first, then have a snack, thereby consuming multiple snacks throughout the interim period, interspersed with excuses. (P2)

Furthermore, underdeveloped fine motor skills, such as inconsistent hand dominance and insufficient grip strength, further contribute to handwriting difficulties:

We appreciate observing children who are unable to write and lack the skill to handle a pencil, as it demonstrates their capacity to apply pressure while using it. Occasionally, there are students who alternate between using their right hand and their left hand for writing. (P7)

### 3.2. Theme 2: Environmental Factors Contributing to Handwriting Improvement

Participants were also questioned about their experiences and thoughts regarding the environment and barriers that could impede the enhancement of handwriting proficiency, and data analysis identified three unique categories: (a) understanding learning behaviors for preparedness, (b) collaboration on handwriting improvement, and (c) environmental support from parents, siblings, and peers. Each of these categories was then examined thoroughly.

#### 3.2.1. Understanding Learning Behaviors for Preparedness

Participants highlighted the importance of understanding children's learning behaviors, including their styles, study habits, and learning environments. Parents are responsible for creating dedicated study spaces at home to minimize distractions.

When the child would study, I would accompany them in a designated study area, as it needed to be more secluded than their bedroom. (P4)

Additionally, teachers also adapt to children's varying learning styles, often incorporating physical activities to help kinesthetic learners focus.

For instance, some children may have a restless nature and struggle to remain still, indicating that their kinesthetic sense is highly noticeable. Prior to proceeding, it is imperative that we grasp the concept at hand. Typically, when dealing with children who have a strong kinesthetic learning style, our approach is to first exhaust their physical energy. Shall we commence with a game of soccer initially? Yes, we began to make them participate in a game of soccer. Subsequently, once they are tired from playing and their energy has been expended, they can then sit and concentrate. (P7)

Furthermore, parents often supplement learning with extracurricular activities to improve handwriting skills.

Kumon and EF for English, He participates in taekwondo and swimming lessons for his physical activity. He enjoys engaging in physical activity. The swimming lessons have been ongoing for 2 years. He has recently resumed his taekwondo training and is now actively participating again. (P4)

Teachers use visual aids, such as alphabet cue cards, to assist children struggling with letter memorization and formation.

Typically, each table in the first grade has a cue card containing the alphabet. (P7)

#### 3.2.2. Collaboration for Handwriting Improvement

Parents and teachers acknowledged the importance of collaboration in supporting children's handwriting development. Parents are encouraged to actively participate in their children's study routines.

It is important to consistently highlight the significance of parental attention and collaboration. Indeed, cooperative commitment implies that you are willing to be inconvenienced. Both parents collaborate to support their children's development, ensuring that they are supervised both at home and school. (P8)

Additionally, warm and supportive parent–child interactions at home accelerate developmental progress compared to children who lack parental engagement.

The influence of parental factors, such as the amount of attention given and the opportunity for practice, can significantly impact a child's development. Additionally, the stimuli provided at home can also have a profound effect. Some parents may be too occupied to dedicate time to instructing their children. Indeed, the child acquires knowledge at school once they have completed their studies at home. It is advantageous for children to have warm and passionate interactions with their parents as it accelerates their developmental progress compared with children whose parents are occupied and lack free time. (P8)

Moreover, peer and sibling interactions also influence handwriting motivation and performance. Positive peer examples inspire children to improve.

At school, focusing on friends as a source of support can lead to a sense of competitiveness or embarrassment, which can hinder one's ability to concentrate on writing. If the outcome is positive, it increases their motivation to learn. (P8)

#### 3.2.3. Environmental Support From Parents, Siblings, and Peers

The interviews revealed that some parents face challenges balancing work and providing academic support to their children.

Indeed, due to my many travels, I lack the presence of someone to assist him in reviewing his teachings, and he is also deprived of the chance to revise or retain what he has learned at school. (P5)

However, parents often dedicate time on weekends for collaborative study sessions.

Not on a daily basis. At least, we make an effort to engage in collaborative study during a particular week. (P3)

Furthermore, the presence of siblings at home can sometimes disrupt study routines.

However, the prevalence of disorders outweighs the amount of knowledge being acquired. There is a greater number of disagreements than seriousness. We are frequently diverted by various disturbances, including weeping, fighting, and the presence of something amiss. Already, that's it. Ended up not learning. (P4)

Similarly, unmotivated peers at school can negatively influence children's interest in handwriting.

However, if he responds inappropriately, it is akin to becoming unresponsive, resembling laziness or a similar state. Therefore, the outcome is contingent upon the child's behavior and characteristics as well. (P8)

## 4. Discussion

This study is aimed at identifying and assessing the characteristics that facilitate and impede the enhancement of children's handwriting, as perceived by parents and teachers. Occupational therapy is defined as “the therapeutic use of everyday life occupations with persons, groups, or populations (i.e., the client) for the purpose of enhancing or enabling participation” [12]. Occupational therapists focus on occupations and factors that disrupt or support these activities, influencing clients' engagement and participation in health-promoting occupations [17]. Understanding the perspectives of parents and teachers regarding factors that improve handwriting abilities may help occupational therapists design effective interventions.

### 4.1. Client Factors Enhancing Handwriting Abilities

Participants highlighted that a child's motivation to learn significantly impacts handwriting improvement. Children who are forced to engage in writing exercises often exhibit perfunctory and disorganized responses. Conversely, when children are motivated and curious, their handwriting becomes more legible. These findings align with Fajariani et al. [18], who emphasized the importance of evaluating and fostering motivation to create effective handwriting programs. Incorporating occupation-based activities, such as traditional games that involve fine motor skills, can enhance children's interest and improve handwriting abilities.

Age readiness was another key factor. Participants noted that children's readiness and confidence in learning depend on their maturity level. Immature children often face cognitive and emotional challenges, leading to difficulties in the classroom. These findings are consistent with Williams et al. [19], who quoted a previous study about child readiness from the National Education Goals Panel [20], which described three components of school readiness: readiness in the child, schools' readiness for children, and family and community support. Age readiness, including physical well-being and sensory motor development, is a critical component of readiness in the child.

Participants also emphasized the significance of fine motor development. Grip strength, grasp patterns, and hand dominance were commonly cited as areas needing improvement. Children who frequently switch hands or exhibit irregular grip patterns as well as difficulties to maintain attention may struggle with handwriting. These findings support Schneider et al. [21], who reported that a dynamic tripod grasp, in-hand manipulation, and well-coordinated use of force positively influence handwriting performance. However, some studies, such as Omar et al. [22], found no significant differences in grip strength between dominant and nondominant hands, highlighting the need for further research.

### 4.2. Environmental Factors Contributing to Handwriting Improvement

Participants noted that understanding and adapting to children's learning preferences positively impact handwriting proficiency. Each child has unique qualities and may exhibit kinesthetic, auditory, or visual learning preferences. Personalized exercises tailored to these preferences can help captivate children's attention and enhance concentration. However, Pashler et al. [23] found limited empirical evidence supporting the meshing hypothesis, which suggests that matching teaching methods to learning styles improves outcomes. Further research involving school-aged children is needed.

Collaboration between parents and professionals, particularly teachers, was also deemed essential. Children who receive additional practice at home often show accelerated developmental progress. Teachers emphasized the importance of parental involvement in creating a supportive learning environment. Warm and affectionate parent–child interactions were associated with positive emotional states and increased receptiveness to lessons, as highlighted by Williams et al. [19], who quoted a previous study from the National Education Goals Panel [20].

Peers and siblings also play a crucial role in handwriting development. Supportive siblings and conscientious peers can inspire children to improve their skills. Conversely, unsupportive environments, including disruptive siblings or unmotivated peers, may hinder progress. Morgan, Spears, and Kaplan [24], cited by Williams et al. [19], noted that strong family and peer bonds are essential for school readiness and related skills, including handwriting.

### 4.3. Implications for Occupational Therapy

The findings of the present qualitative study have significant implications for occupational therapists. First, it provides evidence that motor-related factors as well as other external factors, such as age readiness or environmental support, can affect the enhancement of handwriting abilities. Second, it reveals that occupational therapists can develop an intervention suitable for the children's condition by understanding the perspectives and experiences of parents and teachers. Third, the results of this study also emphasize the importance of collaboration between parents, occupational therapists, and teachers in improving children's handwriting skills. Fourth, it also highlights that siblings and peers significantly affect children's improvement in handwriting performance. Therefore, occupational therapists must consider a school or home program involving siblings and peers for children's socioemotional development, which impacts their performance.

Our findings underscore various factors that are strongly associated with handwriting performance and must be considered by parents and teachers, as well as occupational therapists, when developing handwriting interventions. Our findings may contribute to occupational therapy in both clinical and school-based settings. Nevertheless, further research is warranted to analyze the children's handwriting performance by identifying and assessing the associated components or factors.

### 4.4. Study Limitations

This study has several limitations. Notably, our findings cannot be generalized to the entire population of parents and teachers of children experiencing handwriting difficulties, owing to the limited number of participants in this study. Moreover, cultural and or contextual may influence the findings of this study. Therefore, subsequent studies including caregivers who spend time with the children at home are necessary to gain additional understanding and knowledge and enhance our understanding of children's occupational profiles.

## 5. Conclusions

Handwriting is a multifaceted ability that involves not only the individual but also various surrounding elements. This study examined the experiences and viewpoints of parents and teachers of children with difficulty with handwriting. We examined data pertaining to two main themes: client factors that enhance handwriting abilities and environmental factors that contribute to improving handwriting abilities. Participants highlighted that comprehending the psychological well-being of children, as well as their cognitive and motor abilities, may potentially contribute to the improvement of handwriting skills. Furthermore, they also recognized the significance of collaborating with other professionals to enhance children's handwriting skills. Our results revealed that environmental factors may facilitate or impede handwriting enhancement.

## Figures and Tables

**Table 1:** Semistructured interview questions for parents (in brief).

**S. no.**	**Question content**	**Question type**
1	In your opinion, what is meant by difficulty in handwriting?	Understanding perspective
2	Did you realize that your child has handwriting difficulties? If yes, what did you do to improve your child's handwriting ability?	Understanding experience
3	Do you think your home environment is supportive for your child to learn?	Understanding perspective
4	Do you accompany your child to study at home?	Understanding experience
5	Did your child have siblings to support him/her in studying?	Understanding perspective
6	In your opinion, what factors support children's handwriting ability at home?	Identifying supporting factors
7	In your opinion, what factors hinder children's writing ability at home?	Identifying hindering factors

**Table 2:** Semistructured interview questions for teachers (in brief).

**S. no.**	**Question content**	**Question type**
1	How is the learning process at school?	Understanding experience
2	Do you think the school environment is supportive for students to learn?	Understanding perspective
3	Do you provide additional practice for children who have handwriting difficulties? If yes, what kind of additional practice do you provide?	Understanding experience
4	In your opinion, what factors support children's handwriting ability at school?	Identifying supporting factors
5	In your opinion, what factors hinder children's writing ability at school?	Identifying hindering factors

**Table 3:** Details of themes.

**Themes**	**Categories**	**Subcategories**
Client factors that enhance handwriting abilities	Child's psychological well-being	Regulation of emotions
		Age readiness
	Child's cognitive and motor performance	Memory and attention skills
		Motor skills

Environmental factors that contribute to improving handwriting abilities	Understanding learning behavior for preparedness	Understand child's learning behavior
		Writing exercise
		Setting up a learning environment
	Collaboration on handwriting improvement	Collaboration between parents and professionals
		Building relationship with children
		Sibling and peer support
	Environmental support from parents, siblings, and peers	Opportunity to practice at home with parents
		Reducing interference from siblings and peers

## Data Availability

The data that support the findings of this study are available, but restrictions apply to the availability of these data, which were used with permission for the current study and so are not publicly available. Data are however available from the authors upon reasonable request and with the permission of Dr. Yuko Ito.
